# Immobilization of bacterial mixture of *Klebsiella variicola* FH-1 and *Arthrobacter* sp. NJ-1 enhances the bioremediation of atrazine-polluted soil environments

**DOI:** 10.3389/fmicb.2023.1056264

**Published:** 2023-02-03

**Authors:** Zequn Pan, Yulin Wu, Qianhang Zhai, Yanan Tang, Xuewei Liu, Xuanwei Xu, Shuang Liang, Hao Zhang

**Affiliations:** ^1^College of Plant Protection, Jilin Agricultural University, Changchun, China; ^2^Ginseng and Antler Products Testing Center of the Ministry of Agricultural PRC, Jilin Agricultural University, Changchun, China

**Keywords:** *Klebsiella variicola* FH-1, *Arthrobacter* sp. NJ-1, atrazine, bioremediation, immobilization

## Abstract

In this study, the effects of the immobilized bacterial mixture (IM-FN) of *Arthrobacter* sp. NJ-1 and *Klebsiella variicola* strain FH-1 using sodium alginate-CaCl_2_ on the degradation of atrazine were investigated. The results showed that the optimal ratio of three types of carrier materials (i.e., rice straw powder, rice husk, and wheat bran) was 1:1:1 with the highest adsorption capacity for atrazine (i.e., 3774.47 mg/kg) obtained at 30°C. On day 9, the degradation efficiency of atrazine (50 mg/L) reached 98.23% with cell concentration of 1.6 × 10^8^  cfu/ml at pH 9 and 30°C. The Box–Behnken method was used to further optimize the culture conditions for the degradation of atrazine by the immobilized bacterial mixture. The IM-FN could be reused for 2–3 times with the degradation efficiency of atrazine maintained at 73.0% after being stored for 80 days at 25°C. The population dynamics of IM-FN was explored with the total soil DNA samples specifically analyzed by real-time PCR. In 7 days, the copy numbers of both *PydC* and *estD* genes in the IM-FN were significantly higher than those of bacterial suspensions in the soil. Compared with bacterial suspensions, the IM-FN significantly accelerated the degradation of atrazine (20 mg/kg) in soil with the half-life shortened from 19.80 to 7.96 days. The plant heights of two atrazine-sensitive crops (wheat and soybean) were increased by 14.99 and 64.74%, respectively, in the soil restored by immobilized bacterial mixture, indicating that the IM-FN significantly reduced the phytotoxicity of atrazine on the plants. Our study evidently demonstrated that the IM-FN could significantly increase the degradation of atrazine, providing a potentially effective bioremediation technique for the treatment of atrazine-polluted soil environment and providing experimental support for the wide application of immobilized microorganism technology in agriculture.

## 1. Introduction

As a type of triazine herbicide with selective and translocatable activities, atrazine is primarily used to control grasses and broadleaf weeds in the fields of maize, sorghum, and other crops (Lin et al., 2019; Ma et al., 2019). Due to its high effectiveness, low toxicity, and low cost, atrazine has become one of the most widely applied herbicides in the world (Hansen et al., 2019), severely contaminating both soil and groundwater and damaging sensitive crops due to its high solubility and extended residual characteristics (Zaya et al., 2011; Zhang et al., 2014). The stability of the ecological environment is significantly threatened by the residues of atrazine and its metabolites, interfering the endocrine systems of humans and other animals (Luo et al., 2021). The residue of atrazine stays in soil for a long time, and the degradation half-life of atrazine in soil is 13–261 days (Gao et al., 2018). Many studies have investigated the degradation of atrazine in soil. For example, Wang et al. (2011) studied the degradation of atrazine and residual atrazine in soil by GC–MS, revealing that the half-life of atrazine was 14.1 days. However, the half-life of atrazine in soils of long-term fertilization ranges from 20.6 to 33.2 days. Atrazine is also known with deleterious effects on human and other animals. For example, atrazine could trigger the neurophysiology in common carp (Wang et al., 2018) and historical damage in the liver and tests of *Astyanax altiparanae* (Destro et al., 2021). Furthermore, atrazine has shown an adverse effect on soil microbial community, which severely threatens the sustainability of agricultural soil (Singh et al., 2018). Moreover, studies have shown that exposure to atrazine could reduce the production of testosterone, decrease the sperm motility, and increase the generation of abnormal sperms (Zhu S. H. et al., 2021). Additionally, at the maximum pollutant level (MCL) of atrazine in water (3 μg/L), the atrazine in the soil environments was also toxic to almost all members of the food chain (Rostami et al., 2021). The prolonged residual pollution following the application of atrazine has attracted increasing attention worldwide, promoting the advancements of chemical treatment, adsorption, incineration, and microbiological degradation techniques to remove atrazine from the environments (Getenga et al., 2009), with the microbial remediation rapidly recognized as one of the primary strategies for removing atrazine from the ecological environment due to its advantages of low cost, high effectiveness, and environmental friendliness (Wang et al., 2016).

Microbial degradation is the process characterized by the conversion of complex organic materials into basic inorganic matters by microbes (Miller et al., 2019). To date, the microorganisms that have been isolated to efficiently degrade atrazine include fungi, e.g., white rot fungi, *Trichoderma viride*, and *Rhizopus* (Wolf et al., 2019), bacteria, e.g., *Nocardiaceae* sp. (Plaza et al., 2021), and algae (Desitti et al., 2017; Fernandes et al., 2018). Due to their high adaptability to the environment and convenient cultivation, a variety of bacteria have been widely investigated in the applications of atrazine degradation (Kolekar et al., 2019), including *Arthrobacter* sp. (Zhao et al., 2017a), *Pseudomonas* sp. (Zhao et al., 2017b), *Penicillium* sp. (Yu et al., 2018), and *Bacillus* sp. (Huang et al., 2016). For example, Bhardwaj et al. (2015) isolated the *Pseudomonas* sp. strain EGD-AKN5 to degrade atrazine with the initial concentration of 100 mg/L by 93.30% in 3.6 days, while Kolekar et al. (2014) used *Rhodococcus* sp. BCH2 to degrade atrazine at an initial concentration of 100 mg/L at 75.0% in 7 days. It has been shown that mixed bacterial interactions are more effective in the degradation of atrazine than the single strains (Xu et al., 2019). For example, Jiang et al. (2019) obtained the degradation percentage of atrazine by a single bacterial strain *Arthrobacter* sp. DNS10 at 40.57% in 48 h, which was significantly increased to 99.18% under the co-cultivation with *Enterobacter* sp. P1. Similarly, the mixture of a bacterial strain *Ralstonia pickettii* L2 and a fungal strain *Trichoderma viride* LW-1 enhanced the degradation of chlorobenzene at an initial concentration of 220 mg/L (Cheng et al., 2017). In addition, the study found that Klebsiella has the ability to remediate polluted environment and can be used as bioremediation (Duran-Bedolla et al., 2021). Development of natural polymer-based composite carriers, in combination with nano-Fe_3_O_4_ to form stable agar/carrageenan-Fe_3_O_4_-*Klebsiella pneumoniae* composite beads, which show excellent phenol biodegradation performance (Fang et al., 2021).

The novel soil bacterial strain *K. pneumoniae* GS7-1 was applied for the degradation of zearalenone (Imade et al., 2022). *Klebsiella oxytoca* GS-4-08 has a great potential for treating real nitriles-containing wastewater, and for organic acid production (Liu et al., 2017). Immobilization is a technique to immobilize free cells in a special structural area of the carrier materials and keep them active and reusable. For instance, both *Bacillus subtilis* B99–2 (Ma et al., 2015) and *Bifidobacterium* sp. BB-12 (Fritzen-Freire et al., 2012) were immobilized to improve their stability and biocontrol effects. In general, the immobilized bacteria could not only enhance the remediation of the polluted environments (Table 1), but also gain the advantages of relatively strong stability and reusability (Zhou et al., 2016).

**Table 1:** Atrazine-degrading microorganisms with high degradation efficiency.

Microorganism	Degradation strategy	Atrazine removal efficiency	References
*Rhodobacter sphaeroides* W16	Free bacteria	96.86% in 15 days	Du et al. (2011)
*Arthrobacter* sp. DAT1	Free bacteria	>95% in 3 days	Wang et al. (2013)
*Pseudomonas* sp. ADP	Free bacteria	>79% in 8 days	Lima et al. (2009)
*Klebsiella variicola* FH-1	Free bacteria	81.5% in 11 days	Zhang et al. (2019)
*Klebsiella variicola* FH-1 *and Arthrobacter* sp. NJ-1	Free bacteria	85.6% in 9 days	Gao et al. (2020)
*Arthrobacter* sp. ZXY-2	Immobilized bacteria using corn straw biochar	50 mg/kg in 1 h	Yu et al. (2020)
*Acinetobacter lwoffii* DNS32	Immobilized bacteria using synthesized La^3+^ and polydopamine	100 mg/kg in 48 h	Han et al. (2022)
*Agrobacterium radiobacter* J14a	Immobilized bacteria using phosphorylated-polyvinyl alcohol	>40% in 120 h	Siripattanakul et al. (2008)
*Pseudomonas stutzeri* Y2	Polyvinyl alcohol, sodium immobilized bacteria with alginate, activated carbon, SiO_2_, and nitrogen-doped TiO_2_	100% in 4 days	Zhang et al. (2020)
*Penicillium* sp. yz11-22 N2	Magnetic bionanomaterial including Fe_3_O_4_	91.2% in 120 h	Yu et al. (2018)

In our previous studies, two bacterial strains with high efficiency in atrazine degradation, i.e., *Klebsiella variicola* FH-1 and *Arthrobacter* sp. NJ-1 (Gao et al., 2020), were isolated in the laboratory settings. Furthermore, compared with the separate applications of strains FH-1 and NJ-1, the biodegradability of atrazine by the mixture of these two bacterial strains was significantly improved. The purposes of this study were to generate immobilized bacterial mixture of strains FH-1 and NJ-1 and to characterize the enhanced degradation of atrazine by the immobilized bacterial mixture in soils. The immobilized systems of bacterial suspension were developed with the immobilization matrix composed of rice straw powder, rice husk, wheat bran, and sodium alginate. The optimal culture conditions of atrazine degradation by immobilized bacterial mixture were designed by the Box–Behnken method with the effects of three factors (i.e., temperature, pH level, and initial concentration of atrazine) on atrazine degradation optimized. Furthermore, the reusability and storage stability of the immobilized bacterial mixture were also evaluated. Finally, the colonization dynamics and atrazine removal efficiency by immobilized bacterial mixture in soil were verified, i.e., the phytotoxicity of atrazine on sensitive crops (soybean and wheat) grown in atrazine-polluted soils was attenuated by the treatment of the immobilized bacterial mixture. This study provides a potential and effective bioremediation technology for the treatment of atrazine-contaminated soil environment, and an effective method for maintaining the capacity of immobilized bacterial mixture based on agricultural solid waste, trying to solve the low efficiency and poor environmental adaptability of degrading bacteria in practical application, and providing a potential and promising bioremediation method for improving the genetic stability, high degradation efficiency, and strong adaptability of herbicide-contaminated soil.

## 2. Materials and methods

### 2.1. Chemicals, bacteria, media, and plants

Atrazine (purity: 97.5%) was purchased from TCI Development Co., Ltd. (Shanghai, China) and all other chemicals used in our study were of analytical grade. Both *Klebsiella variicola* strain FH-1 (GenBank accession MH250202) and *Arthrobacter* sp. strain NJ-1 (GenBank accession MH250203) were kept frozen until use at the Pesticide Science Laboratory of Jilin Agricultural University. The rice straw powder, corn straw powder, wheat bran, rice husk, vermiculite, and waste fungal substrate were provided by the Research Center of Mycology, Jilin Agricultural University. The Luria–Bertani (LB) medium contained tryptone 10.0 g/L, yeast powder 5.0 g/L, and NaCl 10.0 g/L, with pH level adjusted to 7.0–7.5. The minimal salt medium contained sucrose 35.3 g/L, NH_4_Cl 10.3 g/L, MgSO_4_ 7 H_2_O 0.4 g/L, KH_2_PO_4_ 0.5 g/L, K_2_HPO_4_ 1.5 g/L, and NaCl 1.0 g/L, with pH level adjusted to 7.0. The meadow black soil samples were collected from the experimental maize field (43°48′49.22″N and 125°25′18.20″E) of Jilin Agricultural University, with pH 6.4, organic matter content of 2.65%, and no application of atrazine. Fresh soil samples were collected from the underground 0–20 cm in depth, dried naturally, and sieved through 40 mesh. The seeds of both wheat (*Triticum aestivum* L. variety “Jimai No. 3”) and soybean (*Glycine max* L. variety “Ji Da No.1”) were obtained from the College of Plant Protection of Jilin Agricultural University and the College of Plant Science of Jilin University, respectively.

### 2.2. Preparation of the bacterial suspensions

Single colonies were picked from FH-1 and NJ-1 solid media and transferred to LB liquid media, incubated at 30°C and 150 r/min for 12 and 18 h, respectively. Then, the samples were centrifuged for 5 min at 7,000 rpm, with the supernatant removed; the bacterial samples of strains FH-1 and NJ-1 were then suspended with sterile water and mixed in a volume ratio of 3:2 (V:V), which was the bacterial suspensions (FN; 4.0 × 10^9^ cfu/ml; Gao et al., 2020).

### 2.3. Screening of carrier materials

A total of six types of carrier materials, i.e., rice straw powder, corn straw powder, wheat bran, rice husk, vermiculite, and waste fungal substrate (sieved through 100 mesh), were weighed 5 g each and loaded into six 250 ml triangular flasks, and sterilized at 121°C for 20 min before cooling and inoculating with 10 ml of FN. The samples were shaken to mix and then air-dried at 30°C to prepare the carrier materials containing bacterial suspensions. The obtained samples were stored at 4°C and 25 ± 5°C (room temperature), respectively. The viable bacterial cell counts in different carrier materials were measured at 2 h, 10 days, 20 days, 40 days, 60 days, and 90 days. Each experiment was repeated three times to determine the optimal carrier materials and storage temperature.

### 2.4. Degradation of atrazine by the bacterial suspensions with varied ratios of carrier materials

The optimal carrier materials screened, i.e., rice straw powder, rice husk, and wheat bran, were mixed with the ratios of 1:1:1, 1:2:1, 1:1:2, and 2:1:1, respectively. A total of 5 g of each mixture of carrier materials were collected and autoclaved at 121°C for 20 min, added into the minimal salt medium containing 50 mg/L atrazine with or without 10 ml FN, respectively, as two treatment groups, shaken and mixed, ventilated, and dried at 30°C. The control experiment contained only atrazine. The concentration of atrazine was determined at 0, 1, 3, 5, 7, and 9 days using high performance liquid chromatogram (HPLC), respectively.

### 2.5. HPLC analysis

The concentration of atrazine was measured using HPLC analysis (Agilent 1260). The wavelength was set to 222 nm on the UV detector using a reverse-phase column C_18_ (4.6 × 250 mm, 5 μm) with a flow rate of 1.0 ml/min (methanol/water = 60/40, v/v), column temperature of 30°C, and an injection volume of 10 μl. The limit of detection for atrazine was 3 ng, and the limits of quantification for atrazine in water and soil samples were 0.03 and 0.02 mg/kg, respectively.

### 2.6. Adsorption of atrazine by the carrier materials

#### 2.6.1. Immobilization of carrier materials

Based on the optimal ratio of carrier materials (M) obtained above, the carrier materials were added with 3% sodium alginate (w/v) to generate the immobilized carrier materials (IM). The mixture was extruded in droplets into 2% sterile calcium chloride solution (w/v) *via* a syringe and cross-linked at 4°C for 24 h to obtain the IM.

#### 2.6.2. Adsorption kinetics

To increase the ionic strength of the solution, a total of 1.1098 g of CaCl_2_ was weighed and dissolved in 1,000 ml of water to prepare the sterile 0.01 mol/L CaCl_2_ solution. A total of 50 ml of 0.01 mol/L CaCl_2_ solution were added to a 250 ml glass vial containing 50 mg/L atrazine to generate three treatments each of 0.2 g of M, 0.2 g of IM, and blank control, respectively. The samples collected in 1, 2, 4, 6, 8, 10, 12, 24, and 48 h were placed in a vibration shaker at 30°C and 150 rpm, filtered with a 0.22 μm filter membrane (Wang et al., 2020), and analyzed by HPLC. All experiments were performed in three replicates with data presented as mean ± standard deviation (SD).

#### 2.6.3. Adsorption isotherms

Based on the results of adsorption kinetics experiment, the adsorption isotherm experiments were performed using the same procedures with initial concentration of atrazine set to 0.5, 5, 10, 20, and 50 mg/L, respectively. The samples were placed in a vibration shaker at 150 rpm for 48 h at three different temperatures of 20, 25, and 30°C to achieve the adsorption equilibrium (Magid et al., 2021). Samples were collected at 0 and 24 h to measure the adsorption. All experiments were performed with three biological replicates.

### 2.7. Preparation of immobilized bacterial mixture

The FN, M, and 3% sodium alginate were mixed and collected in droplets into 2% sterile CaCl_2_ solution (w/v) *via* a syringe and cross-linked at 4°C for 24 h to prepare the immobilized bacterial mixture (IM-FN). The IM-FN obtained was loaded with bacteria of about 1.6 × 10^9^ cells (cfu/g). The IM without bacteria was prepared and stored separately in a refrigerator at 4°C. The morphological features of carrier materials (M), immobilized carrier materials (IM), carrier materials containing bacteria suspensions, and immobilized bacterial mixture (IM-FN) were observed using the scanning electron microscopy (SEM; Zeiss EVO18, Jena, Germany).

### 2.8. Atrazine degradation assays

To determine the optimal culture conditions for the biodegradation of atrazine by IM-FN, the atrazine was incubated in 100 ml of minimal salt medium with varied bacterial cell concentration (i.e., 4.0 × 10^7^, 8.0 × 10^7^, 1.2 × 10^8^, 1.6 × 10^8^, and 2.0 × 10^8^ cfu/ml), atrazine concentration (i.e., 10, 20, 50, 100, and 200 mg/L), pH levels (i.e., 6, 7, 8, 9, and 10), and temperature (i.e., 20, 25, 30, 35, and 40°C), shaken at 150 rpm, with the residual concentration of atrazine and OD_600_ measured in 9 days. The controls contained only atrazine without bacterial inoculation. The degradation efficiency of atrazine by immobilized bacterial mixture and bacterial suspensions was calculated using the degradation efficiency of atrazine of the control as the baseline (Bhatt et al., 2022). Then, the Box–Behnken module of the design-Expert.V8.0.6 was used to design the experiments with the three factors (i.e., temperature, pH level, and initial concentration of atrazine) affecting the degradation of atrazine by IM-FN optimized for response surface analysis (Supplementary Table 2).

Degradation efficiency of atrazine = (C_0_–C_1_)/C_0_, where C_0_ represented the content of atrazine in minimal salt medium without inoculum and C_1_ represented the content of atrazine in minimal salt medium with bacterial suspensions and immobilized bacterial mixture (Zhao et al., 2022).

### 2.9. Stability and reusability of immobilized bacterial mixture

A total of 10 g IM-FN were added to 100 ml minimal salt medium containing 50 mg/L atrazine with the degradation percentage of atrazine measured every 20 days for a total of 120 days. The IM-FN was incubated in 100 ml of minimal salt medium containing 50 mg/L atrazine for 9 days. Then, the IM-FN was removed, rinsed 3–5 times with sterile water, and then added to a new minimal salt medium (100 ml) containing the same concentration of atrazine; this process was repeated five times with the morphology of the IM-FN observed and the degradation percentage of atrazine measured each time.

### 2.10. Cloning of bacterial genes in soils colonized with immobilized bacterial mixture

Both FN and IM-FN were added to plastic pots (9 × 9 cm) filled with soil samples, respectively. The soil samples were collected after 3, 5, 7, 14, and 21 days and stored in the refrigerator at 4°C. Soil DNA extraction kit (SPINeasy DNA Kit for Soil, MP Biomedicals, LLC, Solon, OH, United States) was used to extract total soil DNA based on 1 g of soil sample from different treatments. The Zn^2+^-dependent hydrolase gene *PydC* (Zhang J. P. et al., 2021) and the esterase gene *estD* (Dong, 2019) were cloned from strains FH-1 and NJ-1, respectively.

The standard curve was plotted using the quantitative real-time PCR (qRT-PCR) method for absolute quantification. The total DNA of the soil samples treated with FN and IM-FN was used to perform the qRT-PCR (10 μl reaction) as described in Supplementary material. The total copy numbers of *PydC* and *estD* genes were calculated by the standard curves, with the dynamics of colonization of strains FH-1 and NJ-1 in soil as the functional coordinate and IM-FN expressed in time as horizontal coordinate. The primers used in the PCR are provided in Supplementary Table 1.

### 2.11. Degradation of atrazine in soils by immobilized bacterial mixture

Soil samples were randomly collected underground 0–15 cm, air-dried, and sieved to 2 mm, from the experimental field of Jilin Agricultural University (43°48′49.22″N and 125°25′18.20″E) without atrazine application for at least 2 years to determine the degradation effect of IM-FN on atrazine in the soil. The atrazine solution was added to soil and mixed well with the concentration of atrazine in soil adjusted to 20 mg/kg and the water content adjusted to 20% with sterile water to obtain the contaminated soil sample. A total of four experimental treatments were prepared with: (1) soil spiked with 20 mg/kg atrazine; (2) soil spiked with 20 mg/kg atrazine and FN (1.6 × 10^8^ cfu/g dry soil); (3) soil spiked with 20 mg/kg atrazine and IM-FN (1.6 × 10^8^ cfu/g dry soil); and (4) soil spiked with 20 mg/kg atrazine and IM. Each treatment was incubated at 25°C with 20 g soil samples collected in 0, 1, 3, 7, 14, 21, 28, and 35 days to determine the contents of atrazine in soil.

### 2.12. Detection of atrazine in soil

To determine the residue of atrazine, a total of 20 g of the soil sample were added with 50 ml of acetonitrile and 10 ml of water in a 250 ml Erlenmeyer flask, shaken for 40 min at 30°C, and the filtrate was obtained by vacuum filtration and transferred to a 100 ml stopper cylinder containing ∼8 g of NaCl. The stopper cylinder was shaken up and down 100 times and allowed to stand for 30 min. Again, the stopper cylinder was shaken 150 times and kept still for another 1 h. The top 25 ml of acetonitrile solution was removed and the extracts were evaporated to nearly completely dry at 40°C under reduced pressure using a rotary evaporator. Finally, the evaporated residues were mixed with 1 ml acetonitrile by vortexing for 1 min and then filtered through a PTFE filter (0.22 μm) for HPLC analysis.

### 2.13. Effect of immobilized bacterial mixture on the growth of atrazine-sensitive crops

Three soil treatments were prepared to investigate the alleviation effect of IM-FN on the phytotoxicity of atrazine, including soil sample without atrazine (CK), soil sample with atrazine at concentration of 0.1 mg/kg (A), and soil sample with both atrazine at concentration of 0.1 mg/kg and IM-FN (1.6 × 10^8^ cfu/g soil), incubated for 7 days. The seeds of wheat and soybean were soaked in water, germinated, and selected and sown in each treatment of soil with 10 seeds per pot. The seedlings were grown at 25°C under constant light with the seedling emergence percentage measured on day 3 and the plant height measured every 15 days.

### 2.14. Data analysis

Results of the measurements were given as the average ± standard deviation (SD) of each treatment. The one-way analysis of variance (ANOVA) was performed with DPS software version 7.05 to determine the significant differences between treatments based on a *p* value of 0.05. All experiments were performed in three biological replicates.

## 3. Results

### 3.1. Selection of carrier materials

The effective viable bacterial cell numbers grown with rice straw powder, rice husk, wheat bran, corn straw powder, waste fungal substrate, and vermiculite stored under varied conditions are shown in Table 2. The numbers of viable cells in these carrier materials were in the following order: rice straw powder > rice husk > wheat bran > corn straw powder > abandoned fungus substrate > vermiculite. From 10 to 90 days, varied numbers of adsorbed bacteria were revealed under two culture temperatures, showing higher bacterial growth rate at room temperature (25 ± 5°C) than that of 4°C in 90 days. Based on these results, the top three types of carrier materials (i.e., rice straw powder, rice husk, and wheat bran) with the highest viable cell numbers at room temperature (25 ± 5°C) were selected as the immobilizing materials in this study.

**Table 2:** Effective number of living bacterial cells (×10^6^ cfu/g) grown with different immobilizing materials at different storage temperature and time.

Material	Storage temperature	2 h	10 days	20 days	40 days	60 days	90 days
Rice straw powder	4°C	52	283	4,064	3,552	2,941	1,542
	25 ± 5°C	58	422	3,520	3,782	3,111	2,893
Corn straw powder	4°C	6.5	204	640	124.3	62.1	30.1
	25 ± 5°C	6.3	35.3	236	149	121.3	102.3
Vermiculite	4°C	0.8	5.8	1.2	1.8	–	–
	25 ± 5°C	1.0	70.3	19.8	2.8	0.8	–
Wheat bran	4°C	12	42.4	328	271	254	142
	25 ± 5°C	29	66.7	803	669	304	203
Rice husk	4°C	53	206	4,650	3,200	2,565	1,310
	25 ± 5°C	47.7	355	3,000	3,350	3,030	2,779
Waste fungal substrate	4°C	5.3	9.8	11.7	1.49	0.41	–
	25 ± 5°C	5.6	11.7	12.9	11.1	9.6	9.2

Symbol “–” indicates data not detected.

### 3.2. Determination of the optimal ratios of carrier materials

**Figure 1:**
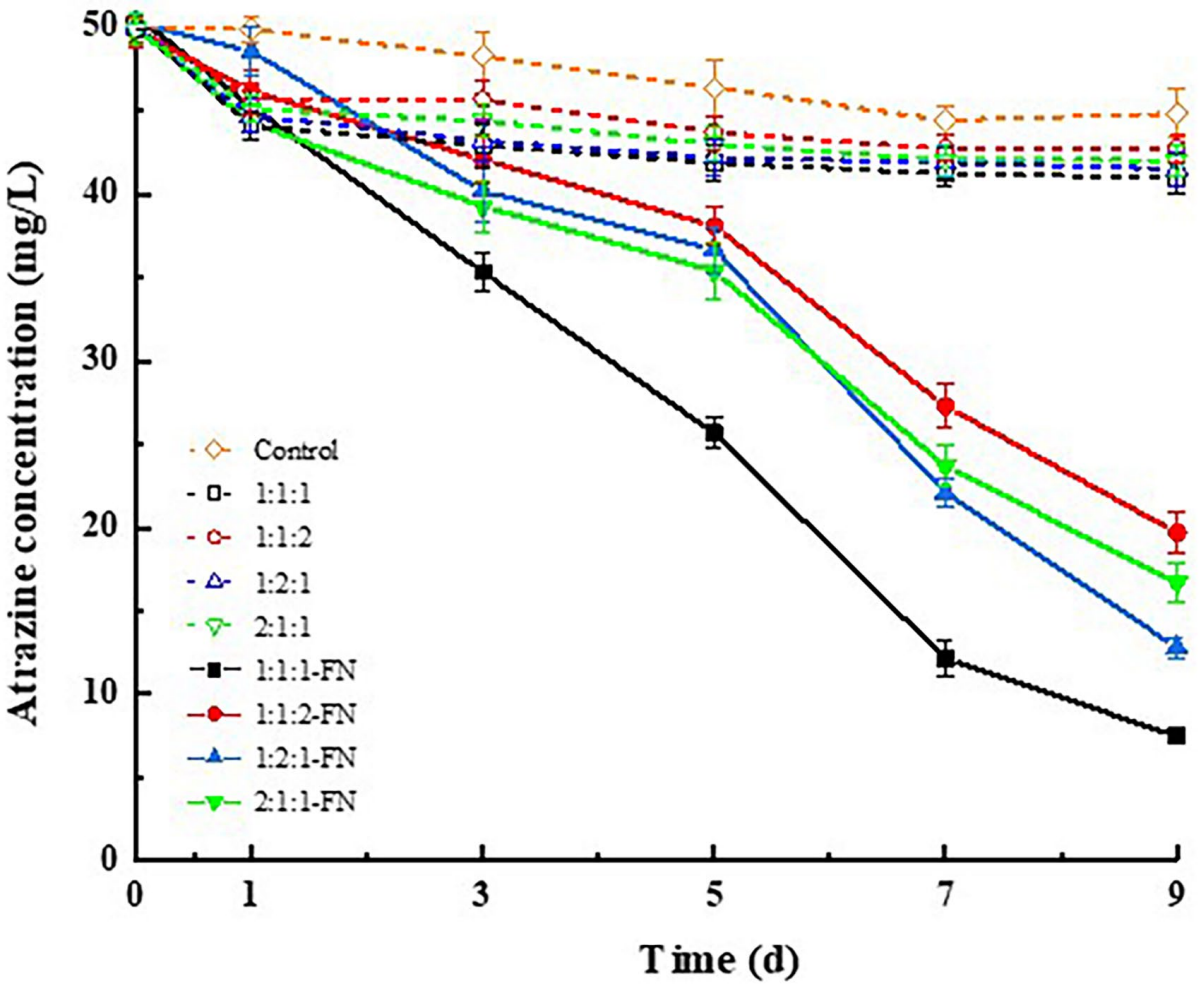
Degradation effect of atrazine by three types of carrier materials (i.e., rice straw powder, rice husk, and wheat bran) at varied ratios with or without the bacterial suspensions (FN).

The degradation of atrazine by carrier materials (i.e., rice straw powder, rice husk, and wheat bran) in four ratios and bacteria is shown in Figure 1. The results showed that the carrier materials alone could adsorb atrazine in the culture medium with varied adsorption capacities. In particular, the highest adsorption efficiencies of atrazine in the culture medium were obtained at 8.65 and 7.49% based on the ratio of these three types of carrier materials (i.e., rice straw powder, rice husk, and wheat bran) at 1:1:1 and 1:2:1, respectively, in comparison with those of 1:1:2 (4.68%) and 2:1:1 (6.31%). With the FN added to the inorganic salt culture medium containing the carrier materials at the ratio 1:1:1 and atrazine (7.50 mg/L), the degradation percentage of atrazine reached the highest 83.29% in 9 days. These results suggested that the highest compatibility was achieved between the carrier materials with rice straw powder, rice husk, and wheat bran mixed in the ratio of 1:1:1 to obtain the highest degradation percentage of atrazine.

### 3.3. Adsorption kinetics and isotherms of immobilized carrier materials

#### 3.3.1. Adsorption kinetics of immobilized carrier materials

**Figure 2:**
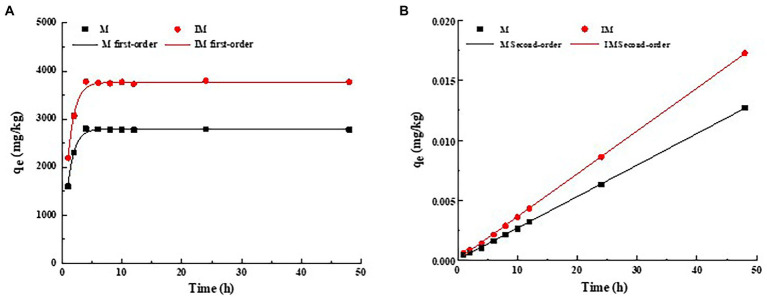
Adsorption pseudo-first-order kinetics **(A)** and pseudo-second-order kinetics plots fitting of *t*/*qt* **(B)** of atrazine on carrier materials (M) and immobilized carrier materials (IM).

In order to explore the adsorption mechanism and absorption percentage of atrazine by the immobilized carrier materials, both pseudo-first-order kinetics and pseudo-second-order kinetics models were used to fit the adsorption results (Figure 2). As shown in Figure 2A, the rapid adsorption occurred in the first 4 h and the pesticide removal percentage reached about 90%. Then, the adsorption was slowed down for about 8 h, and finally reached the predetermined adsorption equilibrium in 24 h. During the rapid adsorption, the adsorption percentage of atrazine by immobilized carrier materials was evidently higher than that of the composite of carrier materials. The maximum adsorption capacities of atrazine by the composite of M and IM reached 2801.12 mg/kg and 3802.281 mg/kg, respectively. The fitting parameters of pseudo-first-order kinetics model (*R*^2^ = 0.98 and 0.98) of the adsorption of atrazine by IM and M were lower than those of pseudo-second-order kinetics model (*R*^2^ = 0.99 and 0.99; Figure 2; Table 3). These results suggested that the pseudo-second-order kinetics model was more suitable for describing the adsorption kinetics of atrazine by M and IM, while the chemical adsorption was probably the main adsorption mode of atrazine by IM and M (Figure 2B).

**Table 3:** Atrazine adsorption kinetics parameters described on carrier materials (M) and immobilized carrier materials (IM) by the pseudo-first-order kinetics model (*R*^2^ = 0.98) and pseudo-second-order kinetics model (*R*^2^ = 0.99).

Sample	First-order kinetics			Second-order kinetics		
	*q_e_*	*K*_1_	*R* ^2^	*q_e_*	*K*_2_	*R* ^2^
M	2794.14 ± 15.07	0.88 ± 0.04	0.98	2801.1 ± 2.28	(29.43 ± 16.59) × 10^−4^	0.99
IM	3764.97 ± 11.68	0.87 ± 0.05	0.98	3802.28 ± 1.63	(22.38 ± 10.23) × 10^−4^	0.99

*q_e_* represents the amount of atrazine (mg/kg) removed at equilibrium; *k*_1_ and *k*_2_ represent the first-order and second-order adsorption percentage constants (1/h), respectively. *R*^2^ represents the fitting parameters of each model.

#### 3.3.2. Adsorption isotherms of immobilized carrier materials

**Figure 3:**
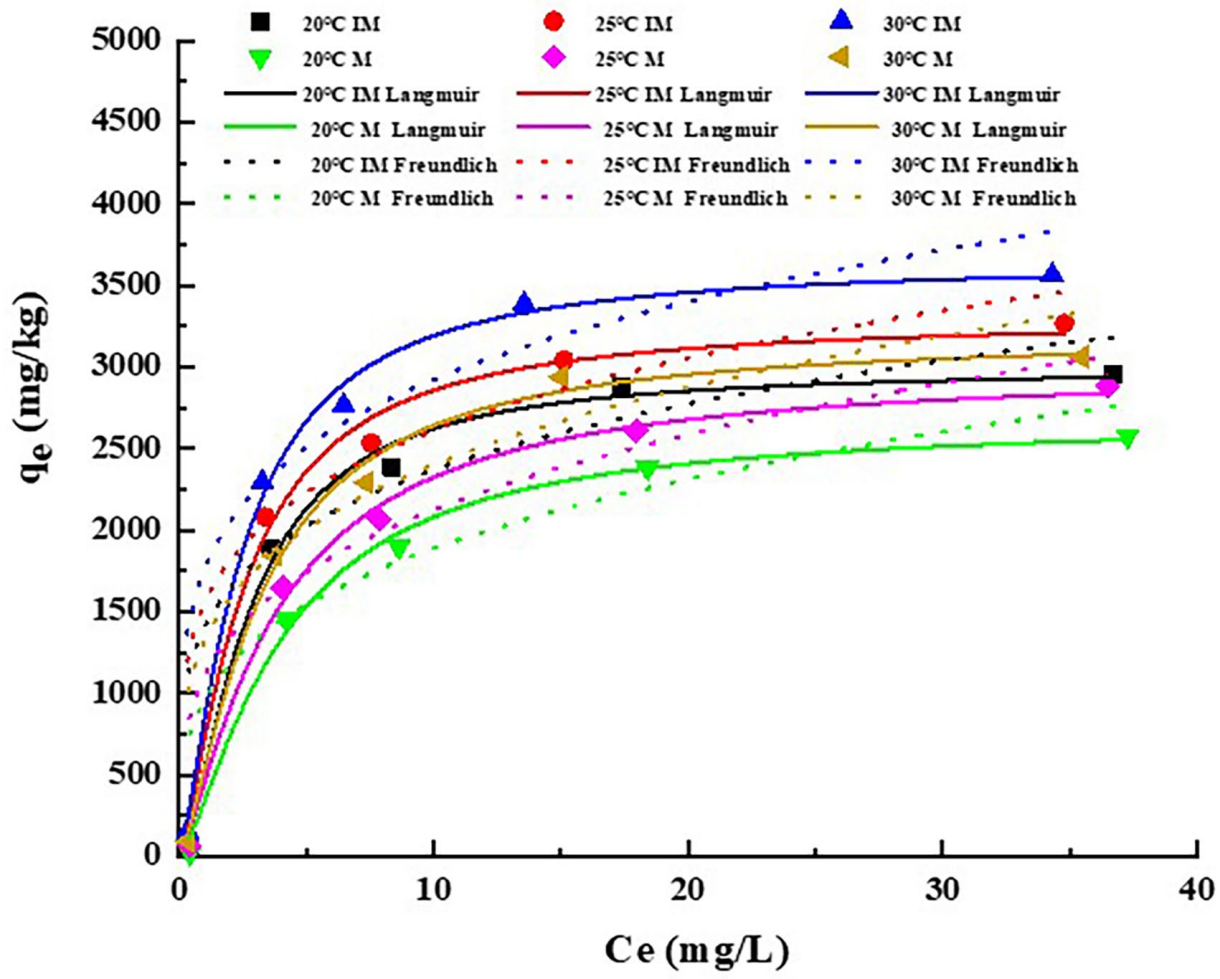
Adsorption isotherms plots of atrazine on carrier materials (M) and immobilized carrier materials (IM). *q_e_* represents the adsorption capacity of M and IM in equilibrium at different temperatures.

The adsorption isotherms of atrazine on the IM at different temperatures are shown in Figure 3. The fitting parameters and coefficients were determined by both Freundlich and Langmuir models (Table 4). The results showed that the Langmuir model fitted better the adsorption process of atrazine on IM and M than the Freundlich model, as indicated by *R*^2^ value greater than 0.97 and the decreased nonlinear coefficient (*n*) with the increase of temperature. Therefore, the Langmuir equation was more suitable for describing the adsorption of atrazine than the Freundlich equation. With the increased concentration of atrazine, the adsorption of atrazine on IM and M was increased rapidly, and then reached a stabilized period. The results showed that different temperatures also affected the adsorption capacity of IM and M of atrazine. With the increase of temperature from 20 to 30°C, the maximum adsorption capacities of atrazine by IM and M were increased from 2994.77 to 3648.42 mg/kg and from 2668.74 to 3190.55 mg/kg, respectively, with the adsorption capacity of IM constantly greater than those of M at different temperatures.

**Table 4:** Atrazine adsorption parameters of the isotherms on carrier materials (M) and immobilized carrier materials (IM) described by the Freundlich and Langmuir equations.

Sample	Langmuir equation				Freundlich equation		
	*T*/*K*	*q_e_*	*K*	*R* ^2^	*K_f_*	*n*	*R* ^2^
M	293	2668.74 ± 157.77	0.15 ± 0.061	0.99	962.24 ± 12.57	0.29 ± 0.004	0.88
	298	2994.88 ± 176.85	0.18 ± 0.061	0.99	1104.85 ± 13.10	0.28 ± 0.004	0.89
	303	3190.55 ± 184.80	0.20 ± 0.08	0.99	1320.78 ± 16.03	0.26 ± 0.04	0.86
IM	293	2994.77 ± 148.38	0.22 ± 0.098	0.98	1404.24 ± 17.67	0.23 ± 0.004	0.81
	298	3298.78 ± 200.15	0.28 ± 0.114	0.98	1550.20 ± 17.45	0.23 ± 0.004	0.84
	303	3648.42 ± 154.27	0.30 ± 0.085	0.99	1763.04 ± 19.40	0.22 ± 0.04	0.83

*q_e_* represents the adsorption amount at equilibrium; *K_f_* represents the Freundlich affinity coefficient [(mg/kg)/(mg/L)]; *n* is the Freundlich constant; *K* represents the adsorption affinity coefficient (L/kg). *R*^2^ represents the fitting parameters of each model.

### 3.4. Morphological observations of immobilized bacterial mixture using scanning electron microscopy

**Figure 4:**
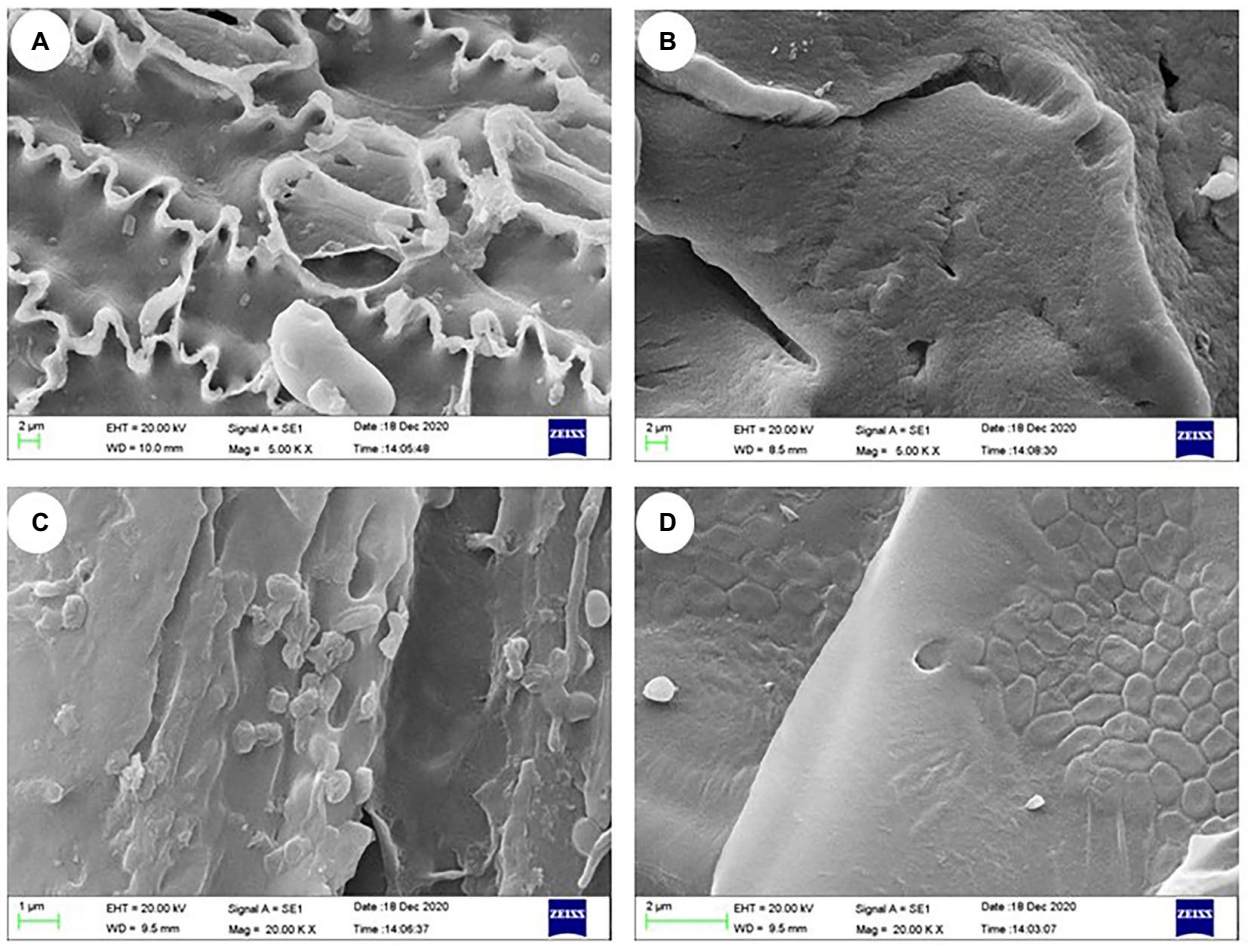
Scanning electron microscope observations of carrier materials **(A)**, immobilized carrier materials **(B)**, carrier materials containing bacterial suspensions **(C)**, and immobilized bacterial mixture (IM-FN) **(D)**.

The morphological features based on SEM observations of the various types are presented in Figure 4. Compared with the carrier materials (Figure 4A), the bacterial suspensions were directly adsorbed on the relatively wrinkled surfaces and the bacteria formed small bubbles (Figure 4C). The carrier materials immobilized with sodium alginate-CaCl_2_ showed dense outer surface with cracks, micropores, and ridges (Figure 4B). Careful examination of cracks and internal structures of the IM-FN revealed bacterial cells embedded in cellulose tubes in the carrier materials (Figure 4D). In our study, rice straw powder, wheat bran, and rice husk with dense fiber structure were used as additives. The SEM images showed that the rice straw powder, wheat bran, and rice hull embedded with sodium alginate contained numerous cellulose tubes, which increased the surface area and porosity, and were conducive to the adhesion of bacteria.

### 3.5. Characteristics of atrazine degradation by immobilized bacterial mixture

**Figure 5:**
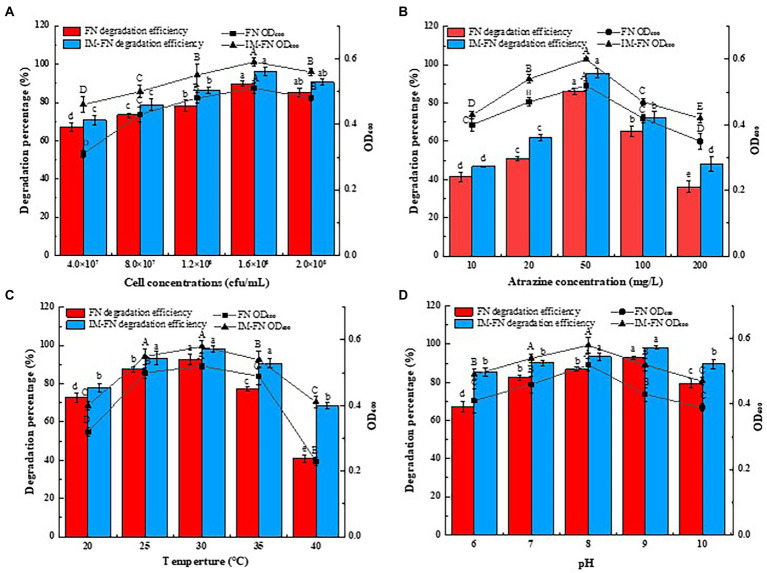
Effects of culture conditions, i.e., bacteria concentration **(A)**, atrazine concentration **(B)**, temperature **(C)**, and pH level **(D)** on degradation of atrazine by bacterial suspensions (FN) and immobilized bacterial mixture (IM-FN). The lowercase letters above the bars indicate significant differences between different treatments at the level of *p* < 0.05; the uppercase letters indicate significant differences between different treatments at the level of *p* < 0.01. Error bars represent standard deviation of triplicate samples.

The effects of varied culture conditions (i.e., pH level, cell concentration of bacteria, temperature, and atrazine concentration) on the degradation of atrazine by IM-FN and FN were investigated to evaluate the practical application potential of IM-FN in the degradation of atrazine. The results showed that the degradation efficiency of atrazine was gradually increased with the increase of bacterial cell concentration (Figure 5A). At the cell concentration of 4.0 × 10^7^ cfu/ml, the degradation efficiency of atrazine by FN and IM-FN were 67.23 and 70.76%, respectively. As the cell concentration was increased to 1.6 × 10^8^ cfu/ml, the highest degradation efficiencies of atrazine by FN and IM-FN reached 89.81 and 96.00% in 9 days, respectively. As the cell concentration was increased to 2.0 × 10^8^ cfu/ml, the degradation efficiency of atrazine by IM-FN was decreased slightly.

Different concentrations of atrazine of 10–200 mg/L significantly affected the degradation of atrazine by FN and IM-FN (Figure 5B). The results showed that the highest degradation efficiency was obtained when the concentration of atrazine was 50 mg/L. In particular, when the concentration of atrazine was 10 mg/L, the degradation efficiencies of atrazine by FN and IM-FN were 41.43 and 46.83%, respectively. With the increase of atrazine concentration, the degradation efficiencies of atrazine by FN and IM-FN were gradually increased. When the concentration of atrazine was increased to 50 mg/L, the degradation efficiency of atrazine by FN and IM-FN reached the highest of 86.13 and 95.70%, respectively, in 9 days. With the increase of atrazine substrate concentration from 10 to 200 mg/L, the degradation efficiency of atrazine by IM-FN was significantly higher than those of FN.

The effects of temperature on the degradation of atrazine are shown in Figure 5C. The highest degradation efficiencies of atrazine were obtained at 92.72 and 98.18% for FN and IM-FN, respectively, at 30°C in 9 days. As the temperature was increased from 20 to 30°C, the degradation efficiencies of atrazine by both IM-FN and FN were increased. As the temperature was increased from 30 to 40°C, the degradation of atrazine was obviously inhibited with the degradation efficiencies of FN and IM-FN obtained at only 40.81 and 68.49%, respectively, at 40°C. Our results revealed poor resistance of FN to temperature in the environment, i.e., when the temperature was higher than 30°C, the atrazine degradation ability of the FN was significantly lower than that of the IM-FN. The effect of pH level also affected the degradation of atrazine by IM-FN and FN (Figure 5D). The highest degradation efficiencies of atrazine by both FN and IM-FN were obtained at pH 7. At pH 9.0, the degradation efficiencies of atrazine by FN and IM-FN were 92.98 and 98.23%, respectively, in 9 days. At pH 6.0, the inhibition of atrazine degradation by FN was higher than that by IM-FN, showing the degradation efficiencies of 67.36 and 85.35%, respectively.

### 3.6. The Box–Behnken analyses

The Box–Behnken design-response surface methodology was used to determine the key factors that affected the degradation percentage of atrazine by the FN and IM-FN. The experiments were performed in minimal salt medium supplemented with atrazine for 9 days. The degradation percentage of atrazine ranging from 65.92 to 98.59% were optimized by three crucial factors, i.e., pH level, initial concentration of atrazine, and temperature. The results of the variance analysis with the best optimization model of atrazine biodegradation are provided in Supplementary Table 3. The regression equation was obtained to represent the atrazine degradation percentage Y with three influencing factors A (pH level), B (temperature), and C (initial concentration of atrazine).


$$\begin{array}{l} Y=97.46+0.2475\;A+0.4663\;B+1.05\;C-0.33\;AB\\ \;\;\;\;\;+0.98\;AC-1.05\;BC-12.45\;A^{2}-14.54\;B^{2}-15.31\;C^{2} \end{array}$$

The magnitude of *F*-value represented the order of influence of different factors on the response value, i.e., A = 0.37, B = 1.32, and C = 6.67, respectively, indicating that the ability of the bacteria to degrade atrazine was influenced by these three factors in the following order: initial concentration of atrazine (C) > temperature (B) > pH level (A) (Supplementary Table 4). The contour plot and the 3D response surface plot showing the optimized culture conditions were evaluated (Supplementary Figure 1). These plots revealed the greater effect of the initial concentration of atrazine and pH level on the degradation of atrazine by IM-FN. The most influential degradation conditions for atrazine degradation by IM-FN were predicted to be pH 9.15, temperature 30.03°C, and initial concentration of atrazine 62.17 mg/kg, with the highest atrazine degradation percentage obtained at 98.19% based on the optimization using the response surface analysis. These predicted conditions were validated by the degradation percentage of atrazine at 98.19 ± 0.5%, indicating that it was appropriate to use the response surface analysis to predict these three factors on the degradation of atrazine by IM-FN.

### 3.7. Stability and reusability of immobilized bacterial mixture

**Figure 6:**
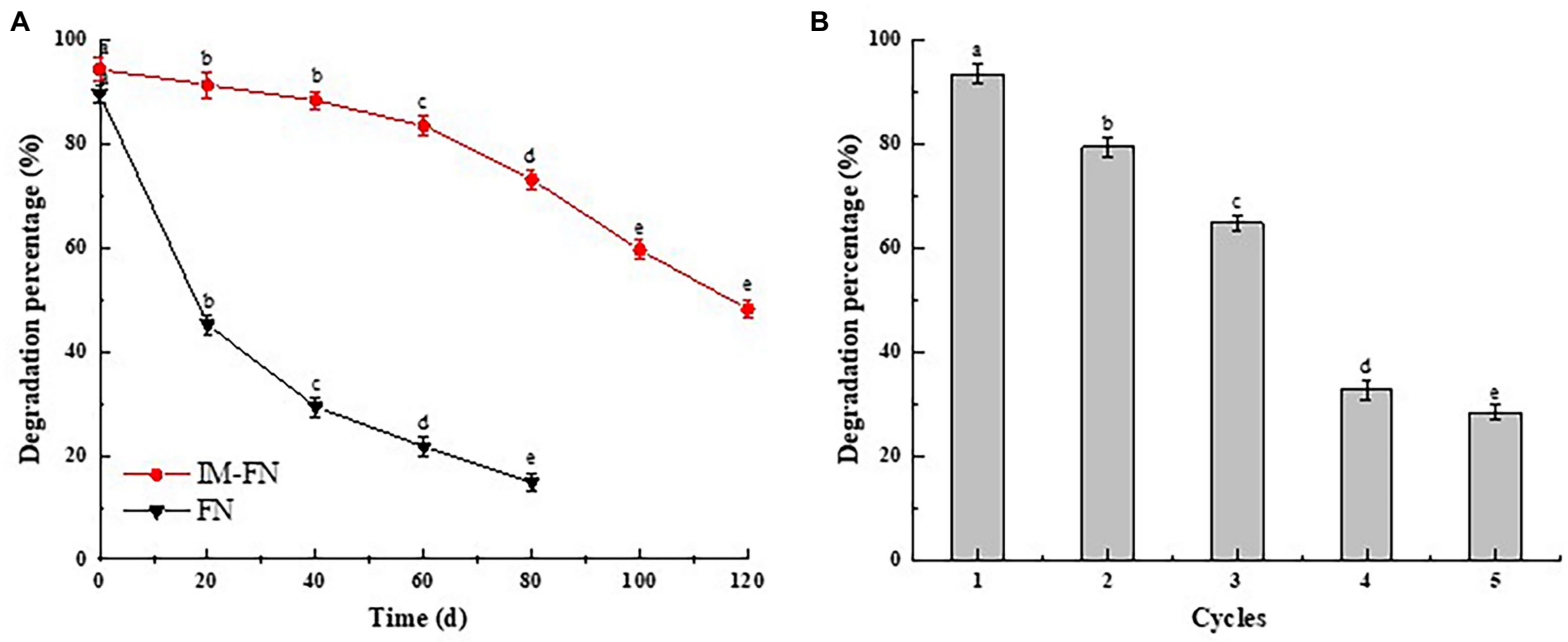
The storage stability **(A)** and reusability **(B)** of immobilized bacterial mixture (IM-FN). Different letters (a, b, c, and d) indicate significant difference between the same treatment at different times based on Duncan’s significant difference test (*p* ≤ 0.05).

The stability of IM-FN was investigated due to its importance in improving the degradation percentage of atrazine (Figure 6A). The results showed that the IM-FN maintained its initial degradation efficiency of 88.25% after being stored at room temperature (25°C) for 40 days, whereas the degradation efficiency of atrazine by FN decreased to 29.36%. In 80 days of storage, the degradation efficiency of atrazine by IM-FN could still reach 73.02%, whereas the degradation of atrazine was not detected by the FN, indicating the high stability of the IM-FN with high degradation efficiency maintained.

The reusability, i.e., the effect of repeated use of IM-FN on atrazine degradation, is shown in Figure 6B. With the increased times of reuse of IM-FN, the degradation percentage of atrazine was gradually decreased. In the third cycle, the degradation efficiency of atrazine still reached 64.80%, whereas in the fourth cycle, the degradation percentage of atrazine was significantly decreased to 32.7%, while the IM-FN began to swell and partially break. These results showed that the IM-FN could be used 2–3 times. To summarize, these results revealed higher reusability and stability of IM-FN than those of FN, providing a potentially feasible bioremediation strategy of the atrazine-polluted soil environments.

### 3.8. Soil colonization by immobilized bacterial mixture

**Figure 7:**
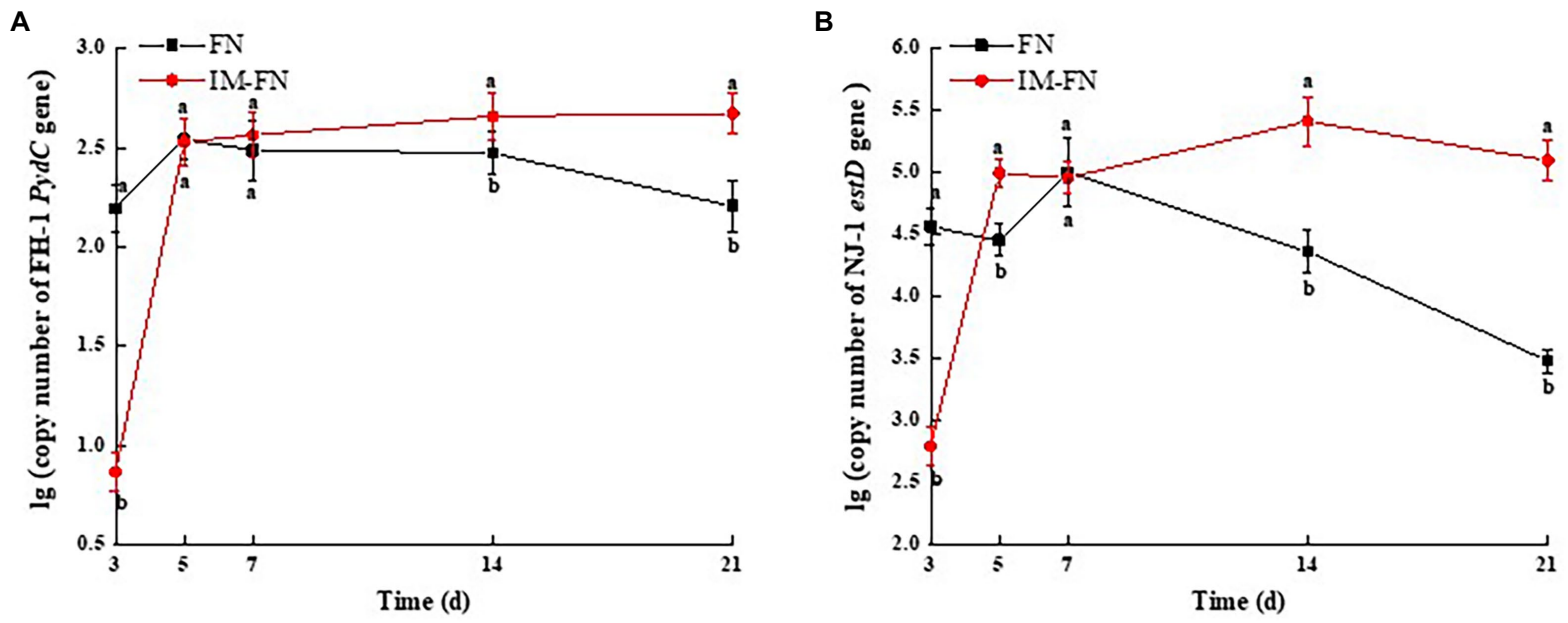
Dynamics of the colonization of strains *Klebsiella variicola* FH-1 and *Arthrobacter* sp. NJ-1 in bacterial suspensions (FN) and immobilized bacterial mixture (IM-FN) in soil for 3, 5, 7, 14, and 21 days. **(A)** The copy number of the *PydC* gene of strain FH-1 in the soils of bacterial suspensions (FN) and immobilized bacterial mixture (IM-FN) at different times. **(B)** The copy number of *estD* gene of strain NJ-1 in the soils of bacterial suspensions (FN) and immobilized bacterial mixture (IM-FN) at different time. Letters a and b indicate significant difference between the same treatment at different times based on Duncan’s significant differences test (*p* ≤ 0.05).

The specificity of strains FH-1 and NJ-1 in FN and IM-FN was detected by PCR using the total DNA of soil samples as template. The results showed that no amplification of the target genes (i.e., Zn^2+^-dependent hydrolase gene *PydC* and esterase gene *estD*) was detected in the total DNA of untreated soil samples (Supplementary Figure 2), while both target genes were amplified in the total DNA of soils supplemented with either FN or IM-FN, respectively (Supplementary Figure 3), indicating the high specificity of strains FH-1 and NJ-1 in soils, and the real-time PCR system based on these two genes could be used to quantify the colonization dynamics of strains FH-1 and NJ-1 in these soil samples. The copy numbers of *PydC* and *estD* genes of strains FH-1 and NJ-1 in the soil treated with FN were significantly higher than those of the IM-FN on the third day, while the copy numbers of these genes of both strains were gradually decreased in 7 days (Figures 7A,B). These results indicated that strains FH-1 and NJ-1 in FN could colonize the soil and become the dominant strains within a short period of time (i.e., less than 3 days), though this effect was not maintained. Furthermore, the detection percentage of target genes of these two bacterial strains in soil treated with IM-FN was significantly lower than that of FN in 3 days. However, the copy numbers of both genes were significantly higher than those of the FN after 7 days, and this effect was maintained until 21 days. These results suggested that IM-FN could enhance the duration of the distribution of both strains in the soil.

### 3.9. Degradation of atrazine by immobilized bacterial mixture in contaminated soils with different treatments

**Figure 8:**
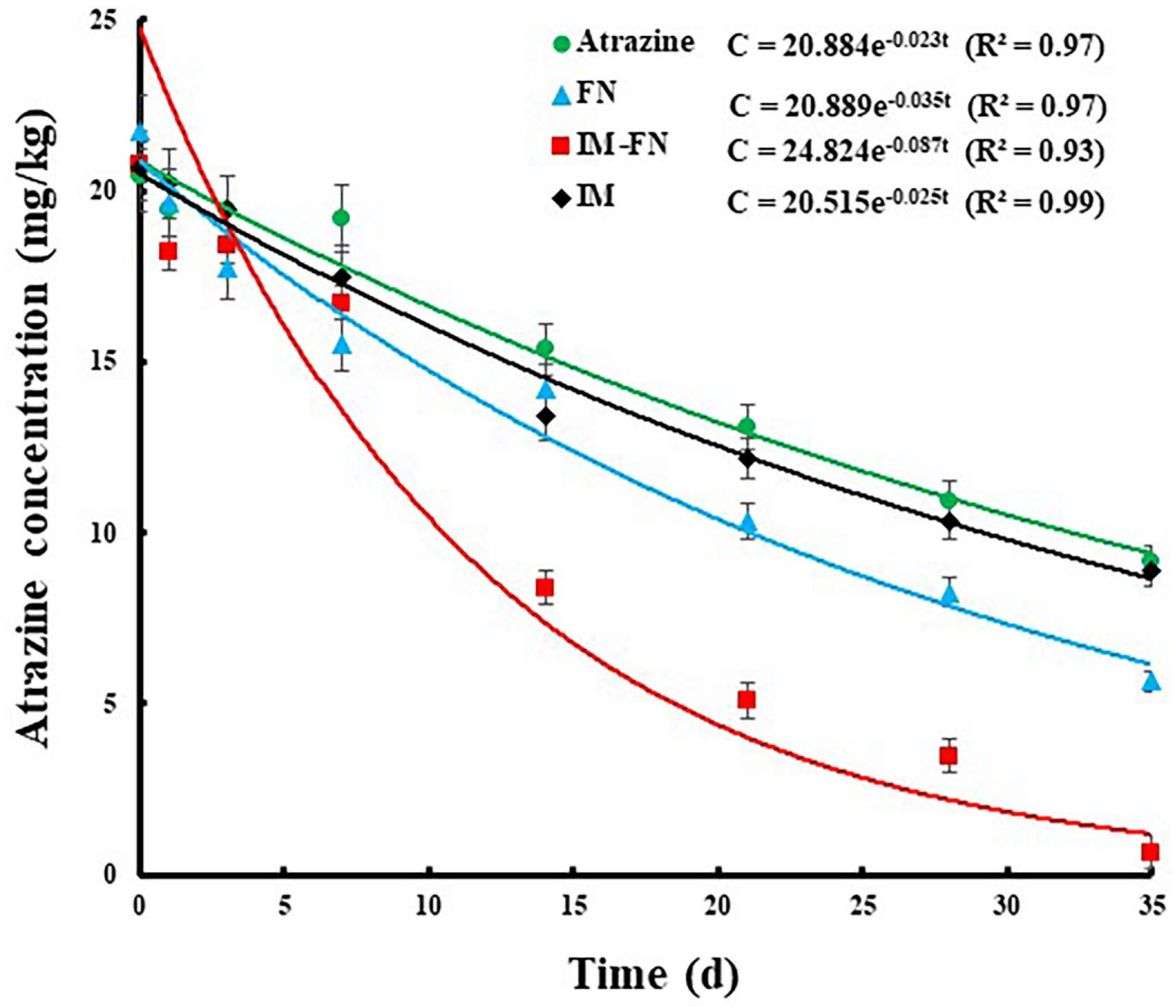
Degradation effect of atrazine in soil treated with atrazine, bacterial suspensions (FN), immobilized bacterial mixture (IM-FN), and immobilized carrier materials (IM).

As shown in Figure 8, the degradation of atrazine in soil under different treatments of atrazine, IM, FN, and IM-FN followed the first-order equations (*R*^2^ > 0.93) of c = 20.884e – 0.023t, c = 20.515e – 0.025t, c = 20.889e – 0.035t, and c = 24.824e – 0.087t, with the degradation half-life of atrazine under the laboratory conditions of 30.13, 27.72, 19.80, and 7.96 days, respectively. These results showed that the introduction of exogenous atrazine-degrading bacteria and IM-FN into the soil significantly promoted the dissipation of atrazine and shortened the half-life of atrazine. In 35 days, the degradation efficiencies of atrazine by IM-FN and FN in soil reached 96.84 and 73.97%, respectively, Our study showed that in 35 days, the degradation efficiency of atrazine in soil by FN reached only 73.97%, suggesting that the degradation of atrazine by direct spraying of bacterial suspension could be easily affected by the uncertain characteristics of soil matrix. However, compared with the FN, the IM-FN showed high applicability in the atrazine-polluted soils and increased degradation efficiency by 22.87% in 35 days, evidently indicating that the combination of atrazine-degrading bacteria and immobilizing materials could more effectively remediate the atrazine-contaminated soil environments.

### 3.10. Effect of immobilized bacterial mixture on the growth of wheat and soybean plants

**Figure 9:**
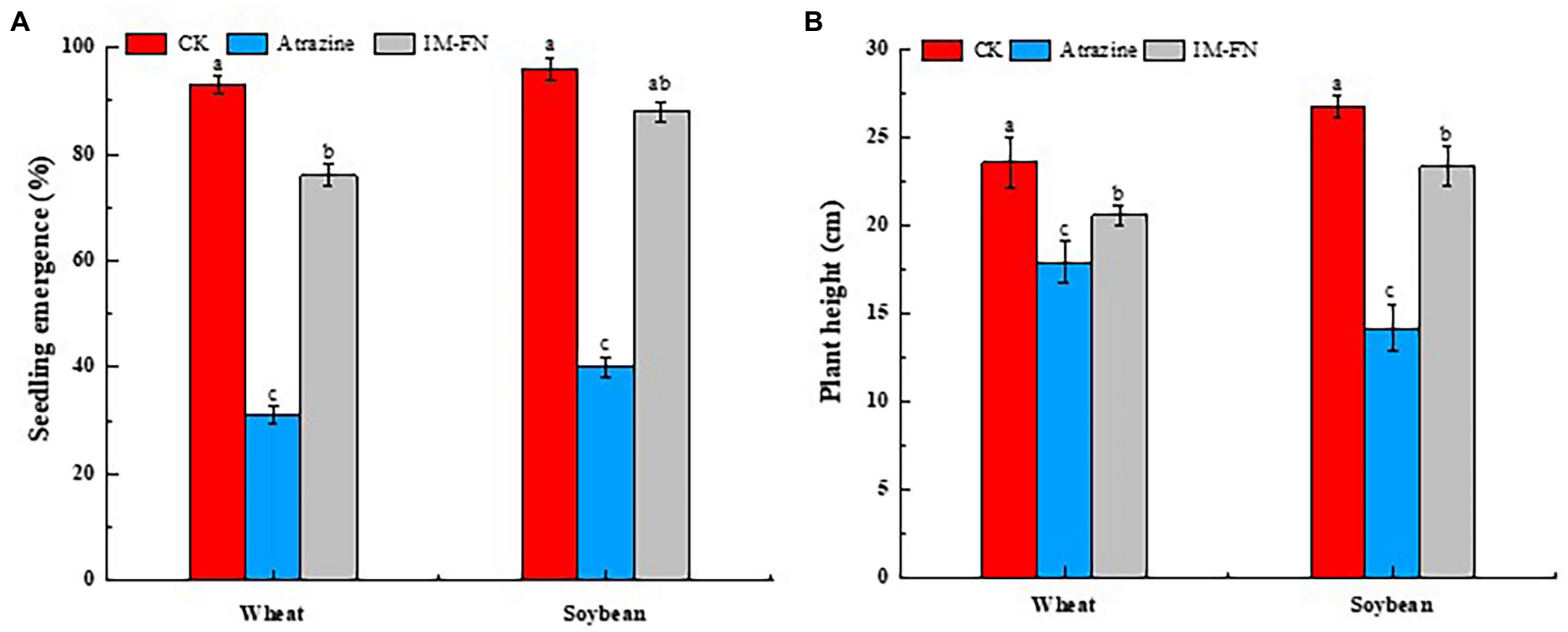
Seedling emergence percentage in 3 days **(A)** and plant height in 15 days **(B)** of wheat and soybean plants grown in soils treated with immobilized bacterial mixture (IM-FN). Different letters (a, b, and c) indicate significant differences between different treatments at the level of *p* < 0.05.

The application of IM-FN in soil showed significant effects on seedling emergence and plant height of both wheat and soybean (Figures 9A,B). With the application of both atrazine at a concentration of 0.10 mg/kg and 10 g of IM-FN in soil, the seedling emergence percentage of wheat and soybean were 76.00 and 88.00%, and the plant heights were increased by 14.99 and 64.74%, respectively, compared with the group in the soil without IM-FN. These results indicated that the addition of IM-FN could promote the degradation of residual atrazine in the soil to reduce the harmful effects of the phytotoxicity of atrazine on wheat and soybean plants.

## 4. Discussion

### 4.1. Adsorption effect of immobilized carrier materials on atrazine

Our results showed that in 4 h, the atrazine was quickly adsorbed by both IM and M. This was probably because the high concentration of atrazine at the interface between both M and IM and the bacterial solution promoted a strong driving force of mass transfer, ultimately causing atrazine to quickly occupy the adsorption sites, which allowed the rapid physical adsorption of atrazine in a short time. These results were consistent with those reported previously (Ren et al., 2022). With the slow saturation of the active sites, the resistance was gradually increased, which was due to the fact that the immobilization technology improved the mechanical strength and chemical stability of the carrier materials at different degrees and the pore structure of the carrier materials, ultimately enhancing the total adsorption capacity of atrazine (Yu et al., 2018). Furthermore, the pore volume of M was smaller and atrazine could reach the adsorption equilibrium quickly, though the adsorption capacity was relatively limited, which as consistent with the results reported previously (Hu et al., 2021). Overall, our results revealed a stronger ability to adsorb atrazine by IM than M.

Both Langmuir and Freundlich models were used to simulate the adsorption process of atrazine, showing that Langmuir model was more consistent and suitable than Freundlich model to describe the adsorption process. The results showed that the temperature increase in a certain range could enhance the adsorption capacity of atrazine by carrier materials. Studies have shown that the adsorption of atrazine by IM and M is mainly the monolayer adsorption of the chemical adsorption (Macías-García et al., 2017). With the adsorption capacity of atrazine by IM constantly higher than that of M and increased with the increase of temperature. This was probably because that the increase of temperature caused a positive effect of carrier materials on the adsorption of atrazine, indicating that the adsorption of atrazine was an endothermic process. Furthermore, the increase of pore size of composite carrier materials at high temperature was probably one of the reasons for the increased adsorption capacity of adsorbent, as reported previously (Ali et al., 2022).

### 4.2. Scanning electron microscopy observation of immobilized bacterial mixture

Compared with other types of carrier materials such as polyvinyl alcohol used in bioremediation technology, alginate generally shows smaller mass transfer resistance (Zhang et al., 2022). The carboxyl group of sodium alginate could exchange with Ca^2+^ and cross-link to form calcium alginate beads. Studies have shown that the SEM observations suggested that adding specific additives to sodium alginate beads was beneficial to improve the biosorption performance of alginate beads (Jampala et al., 2016), with straw, sawdust, and sugarcane generally used as additives (de Castro et al., 2001). Studies have shown that hollow cellulose tubes contained in wheat bran contribute to the diffusion of both oxygen and substrate, ultimately maintaining the microbial activity (Li Y. et al., 2022). The bacterial growth in storage strongly supported the hypothesis that IM and its organic components could provide abundant carbon sources for bacterial colonization. The appearance of voids and folds on the outer surface of microspheres was beneficial to the adsorption of pollutants and the transfer of oxygen and nutrients from IM-FN, which enhanced the growth of microorganisms, as reported previously (Mannacharaju et al., 2021). Studies have shown that the extremely wrinkled structure provides a natural shelter for bacteria to inhabit, which could protect the selected bacteria from the direct competition with other bacteria and the threat of atrazine (Wei et al., 2020). Therefore, these studies suggested that the wrinkled structure could improve the carrier porosity and specific surface area, thus reducing the diffusion resistance and facilitating the transportation of oxygen and nutrients, ultimately accelerating the degradation of atrazine.

### 4.3. Effects of different culture conditions on degradation of atrazine by immobilized bacterial mixture and the optimization of culture conditions

The biodegradation of atrazine by FN and IM-FN was affected by pH level, cell concentration of bacteria, temperature, and atrazine concentration. Among these factors, the initial atrazine concentration was a particularly important factor based on the results of optimization experiments. Although atrazine could be effectively degraded by both FN and IM-FN, atrazine has been revealed with certain toxicity. With the increase of atrazine concentration, its degradation activity was significantly decreased. However, compared with FN, the IM-FN could still maintain a high degradation efficiency under long storage, probably due to the fact that the IM provided a relatively stable microenvironment for the bacteria, while this structure with a tight outer layer and a gradually loose inner surface provided a strong buffer capacity for alleviating the atrazine toxicity, thus protecting the immobilized microorganisms from being inactivated. These results were consistent with those previously reported (Khosla et al., 2017). Therefore, with the diffusion of atrazine to IM-FN, the concentration of atrazine was gradually decreased, slowing down the stress of atrazine on the atrazine-degrading bacteria. The temperature fluctuation over a certain range may increase or decrease the removal percentage of atrazine by affecting the biological activity of bacteria, thus reducing the adsorption of atrazine on cells. The low temperature is generally not conducive to the growth of bacteria, while under high temperature, the bacteria grow fast and quickly entered the decline stage with the enzymes in the bacteria cells inactivated, resulting in the decreased metabolic capacity and growth inhibition of the bacteria (Qi et al., 2020). The IM-FN still maintained a relatively high degradation efficiency at high temperature, probably due to the protection of immobilizing materials, which minimized or tolerated the influence of temperature changes (Li et al., 2020). Proper bacterial inoculation was beneficial to the degradation of atrazine. With the small inoculation, the bacteria grew slowly with low cell vitality, whereas the large inoculation shortened the growth cycle of the bacteria, resulting in insufficient substrate for bacterial growth and metabolism (Cai et al., 2021). The pH level also affected the degradation of atrazine by FN and IM-FN. The results showed that the IM-FN could degrade atrazine under a wide range of pH levels, i.e., 6.0–10.0, with higher degradation efficiencies obtained in neutral or alkaline conditions than those in acidic conditions. Furthermore, the degradation efficiency of atrazine by FN under highly acidic or alkaline conditions was significantly lower than that of IM-FN, which was probably due to the protection of the bacteria by the IM, providing a stable environment for microorganisms and to keep their cell activity, thus playing an important role even in the adverse environmental conditions (Jiang et al., 2022). In our study, the response surface methodology was used to optimize the culture conditions of atrazine degradation by IM-FN. The Box–Behnken model is commonly used to determine the relationship between responses and variables and to calculate the optimal responses (Abdollahi et al., 2012). For example, the Box–Behnken design-RSM was used to optimize the culture conditions of *Bacillus* sp. FA3 in the degradation of fipronil (Bhatt et al., 2021). Indeed, there are many factors that affect the degradation of atrazine. The three factors chosen in our study are the most influential effect on the degradation of atrazine (Guo et al., 2022). Furthermore, the selection of these three factors is verified by the findings revealed in our study, i.e., results presented on Figure 5A showed that the bacterial inoculation amount had trivial effect on the degradation of atrazine. Therefore, we chose these three factors (i.e., temperature, pH level, and initial concentration of atrazine) to optimize the degradation conditions.

### 4.4. Stability and reusability of immobilized bacterial mixture

Our studies revealed higher reusability and stability in IM-FN than those of FN, mainly because the IM and sodium alginate provided sufficient space for bacterial growth (Liu et al., 2012), which, in turn, improved the mechanical properties and stability of the fixed materials, as reported previously (Chen et al., 2020). Furthermore, our studies revealed that the immobilization system showed high durability and compatibility with free cells in water, which improved the adsorption capacity and biodegradation capacity of IM-FN, delayed the contact between microbial cells and the environment, and protected them from adverse environment (Zhang et al., 2020). The IM-FN achieved strong reusability, indicating that it was feasible to remediate the polluted environment by this methodology. Furthermore, compared with free cells, one of the advantages of using immobilized cells to remove atrazine was the reusability of the materials for multiple rounds of application. Meanwhile, the recycling of IM-FN could reduce the cost of manufacturing transportation materials and IM-FN and further promote the popularization and application of this technology.

### 4.5. Colonization dynamics of strain FH-1 and NJ-1 of immobilized bacterial mixture in soils

Previous studies showed that the total copy number of both bacterial strains of *Pseudomonas protegens* FD6 and *Bacillus subtilis* NCD-2 in the soil was gradually reduced with time in 14 days after inoculation as detected by qRT-PCR (Zhang Q. X. et al., 2021). These results were consistent with the finding revealed in our study, indicating that most of the exogenously added bacteria could colonize the soil for a long time. Surprisingly, our results revealed the improved colonization of IM-FN in soil, which was probably due to the gradual release of the microorganisms, whereas the FN of strains FH-1 and NJ-1 could not persistently colonize the soil. These results were consistent with those previously reported (Dos-Santos et al., 2020). The ability of atrazine-degrading bacteria to persistently colonize the soil was an important factor determining their degradation effect. For example, studies have shown that *Mycolicibacterium* sp. Pyr9 colonized the soil for a long time and significantly enhanced the degradation and reduced the content and accumulation of pyrene in white clover (Yang et al., 2021). The joint bioremediation effects of bensulfuron-methyl-degrading strain *Hansschlegelia zhihuaiae* S113 and arbuscular mycorrhizal fungi on bensulfuron-methyl contaminated soil were evaluated (Qian et al., 2022). The results showed that arbuscular mycorrhizal fungi enhanced the colonization of strain S113 in maize rhizosphere and the coexistence of arbuscular mycorrhizal fungi in rhizosphere soil, while strain S113 could remove 3 mg/kg BSM from corn rhizosphere soil within 12 days. Our results suggested that IM-FN could effectively enhance the colonization of strains FH-1 and NJ-1 in soil and promote the degradation ability of atrazine by FN.

### 4.6. Remediation of atrazine-contaminated soil and the enhanced growth of sensitive crops by immobilized bacterial mixture

Studies have shown that the FN introduced into atrazine-contaminated soil is generally vulnerable with poor adaptability due to the influence of soil characteristics and competition with the indigenous communities (Zhu C. Y. et al., 2021). Our results show that the removal efficiency of immobilized microorganisms in soil is higher than that of free cells, suggesting that the IM-FN showed significant potential in remediating the atrazine-polluted soil environment. Li J. Y. et al. (2022) have isolated a novel bacterial strain, *Stenotrophomonas acidophilia* Y4B, to degrade glyphosate and its main metabolite, aminomethylphosphonic acid (AMPA). Strain Y4B degraded glyphosate in a wide concentration range (50–800 mg/L) to achieve high degradation efficiency over 98% in 72 h. Furthermore, strain Y4B showed strong competitiveness to significantly accelerate the degradation rate of glyphosate in both sterile and nonsterile soils to 71.93 and 89.81% (about 400 mg/kg), respectively. Moreover, the immobilized cells of Y4B showed a higher degradation effect on glyphosate than the free bacterial cells. Carrier materials used for microbial immobilization can play a buffering role between soil environment and microorganisms, and protect microbial cells from the harsh and changeable conditions of soil matrix (Girijan and Kumar, 2019). Walha et al. (2022) have comprehensively investigated the application of biochar as a type of carrier material to immobilize metribuzin-degrading bacterial colonies composed of four bacterial strains, which are used to repair metribuzin-contaminated soil and restore soil bacterial communities. The results revealed significantly higher metribuzin remediation in the bacterial alliance immobilized on biochar compared with the soil enhanced by the immobilized bacterial alliance. Furthermore, compared with the degradation rate of 0.010 Kd^−1^ and the half-life of 68 days in the treatment with non-immobilized bacteria alliance, the immobilization of MB3R on biochar resulted in significantly higher MB degradation rate (0.017 Kd^−1^) and shortened half-life (40 days). These alleviation effects of IM-FN were validated by the enhanced growth of wheat and soybean plants grown in soils treated with both atrazine and IM-FN. These results revealed the deleterious effects of atrazine on the physiological activities in soybean and wheat plants, while the alleviation effects of IM-FN on the phytotoxicity of atrazine could be explained in two ways. First, the IM-FN improved the environmental conditions of two atrazine-sensitive crops by removing atrazine in soil or reducing its concentration. Second, the IM-FN played an important role in promoting plant growth and antioxidant activity, thus enhancing the resistance of plants to atrazine. These results were consistent with those previously reported (Pan et al., 2017).

## 5. Conclusion

In this study, we used rice straw powder, rice husk, and wheat bran with the ratio of 1:1:1 and bacterial strains FH-1 and NJ-1 for immobilization with sodium alginate. Our results showed that IM improved the adsorption capacity for atrazine. The degradation performance of IM-FN was further optimized using the Box–Behnken method. Our results showed that compared with FN, the IM-FN showed not only high degradation ability, but also improved stability and reusability as well as enhanced soil colonization ability, indicating that the IM-FN could improve the soil colonization and increase the colonization time of strains FH-1 and NJ-1, ultimately promoting their atrazine degradation ability and the agricultural application of both bacterial strains in remediating of atrazine-polluted soil environments. Furthermore, the immobilization of both bacterial strains significantly improved the degradation ability of atrazine. Compared with FN, the IM-FN significantly accelerated the degradation of atrazine in soil. Moreover, the alleviation effects of IM-FN on the phytotoxicity of atrazine were verified by the enhanced growth of atrazine-sensitive crop plants, showing significant potential of an effective bioremediation technique for the treatment of atrazine-polluted soil environments. In particular, the results of this study are helpful to optimize the bioremediation of atrazine-polluted environments by immobilized microorganism technology, provide an improved understanding of the removal mechanism of atrazine by the immobilized microorganism technology, and effectively treat micro-pollutants in agricultural soils. It is noted that in the future research, it would be important to investigate the influence of immobilized bacteria on the structural composition of the microbiota and the functions of microorganisms in atrazine-contaminated soils and to provide new insights for the practical application of functional microorganisms in atrazine soil remediation.

## Data availability statement

The original contributions presented in the study are included in the article/Supplementary material, further inquiries can be directed to the corresponding authors.

## Author contributions

ZP and YW made the experiences in the article. ZP, YW, and QZ wrote the manuscript. XL, XX, and YT performed the statistical analysis. SL and HZ contributed to the design of the study and corrected the article. All authors contributed to the article and approved the submitted version.

## Funding

This work was financially supported by the Natural Science Foundation Project of the Science and Technology Department of Jilin Province, China (20200201215JC), Project of the Science and Technology Development Plan of Changchun, China (21ZGN12), the “13th Five-Year” Science and Technology Research Project of Education Department of Jilin Province, China (JJKH20200346KJ), and National College Students’ Innovation and Entrepreneurship Training Program, China (202110193013).

## Conflict of interest

The authors declare that the research was conducted in the absence of any commercial or financial relationships that could be construed as a potential conflict of interest.

## Publisher’s note

All claims expressed in this article are solely those of the authors and do not necessarily represent those of their affiliated organizations, or those of the publisher, the editors and the reviewers. Any product that may be evaluated in this article, or claim that may be made by its manufacturer, is not guaranteed or endorsed by the publisher.
