# Supplementary material for: Immobilization of bacterial mixture of Klebsiella variicola FH-1 and Arthrobacter sp. NJ-1 enhances the bioremediation of atrazine-polluted soil environments
Source: Front Microbiol. 2023 Feb 3;14:1056264. doi: 10.3389/fmicb.2023.1056264 (PMC9937183; doi:10.3389/fmicb.2023.1056264)
Supplement: Supplementary file 1 [file Data_Sheet_1.docx]

Supplementary Materials

# PCR and qRT-PCR Analyses

The PCR was performed in the following conditions: pre-denaturation at 94 ℃ for 5 min, followed a total of 30 cycles of “denaturation at 94 °C for 1 min, annealing at 55 °C for 1 min, and extension at 72 °C for 1.5 min” and the final extension at 72 °C for 8 min. The PCR-amplified products of the target genes were run on the agarose gel, recovered, and shipped to Shanghai Biotech (Shanghai, China) for sequencing. The total DNA of strains FH-1 and NJ-1 was extracted using the bacterial DNA extraction kit (Tiangen Biochemical Technology Co., Ltd., Beijing, China) and diluted 5 gradients in 10-fold order to be used as templates for the qRT-PCR analysis. The qRT-PCR amplification was performed using 1 μL of DNA dilution as the template, and the range of the optimum standard concentration was determined by taking the logarithm of the DNA mass concentration (ρDNA) with a base of 10, i.e., lg(ρDNA), as the horizontal coordinate and the cycle threshold of detection (Ct) as the vertical coordinate, with 3 replicates for each dilution gradient. The range of the optimum standard concentration was determined to describe the linear relationship between the lg(copy number of the standard concentration) and the number of cycles to reach the threshold (Ct value). Each qRT-PCR analysis contained forward primer 0.2 μL, reverse primer 0.2 μL, template DNA 1 μL, 2X TaqMan Fast qRT-PCR Master Mix 5 μL, and 3.6 μL ddH_2_O. The qRT-PCR program was as follows: 95 °C for 30 sec, followed by 35 cycles of “95 °C for 5 sec and 62 °C for 31 sec,” 95 °C for 15 sec, 60 °C for 60 sec, and 95 °C for 15 sec.

**Table 1.** The primers and their sequences used in PCR and qRT-PCR analyses for qualitative determination of immobilized bacterial mixture (IM-FN) and bacterial suspensions (FN) in soil and dynamics of the colonization of *Klebsiella variicola* FH-1 and *Arthrobacter* sp. NJ-1. “F” and “R” represent forward and reverse primers, respectively.

| **Gene** | **Primers for PCR** | **Primers for qRT-PCR** |
| --- | --- | --- |
| *PydC* | F: 5’-CATGAGTCATTATCTGCAGAT-3’  R: 5’-GCGCCCAGGCGTATGGACTAA-3’ | F: 5’-CGCTAAGCCTTGGTGGTAATCAGG-3’  R: 5’-CTTATGTCTGGCGGAGCA GCAATT-3’ |
| *estD* | F: 5’-CACCATGAGCACCGCGACGC-3’  R: 5’-CGGTTCCTCGCGCGGTAG-3’ | F: 5’-CGCCAGCGGTCTCAAAGTACAG-3’  R: 5'-CATCAT CGG GTCACGGAAGAGTTC -3' |

# Calculation of Adsorption Kinetics and Isotherms

The amount of atrazine adsorbed onto carrier materials and immobilized carrier material was calculated as follows:

$q_{t}=\frac{\left( C_{i}-C_{e} \right)V}{m}$, (1)

where q_t_ represents the amount of atrazine removed per kilogram of carrier material and immobilized carrier material (mg/kg) at time *t*, *V* represents the volume (L) of the carrier material and immobilized carrier material, *C_e_* represents the equilibrium concentration of atrazine (mg/L), *C_i_* represents the initial atrazine concentration (mg/L), and m represents the mass of carrier material and immobilized carrier material (kg).

The adsorption kinetics experiments were performed based on the pseudo first-order kinetics [Eq. (2)] and pseudo second-order kinetics [Eq. (3)] as follows:

$q_{t}=q_{e}\left( 1-e^{-k_{1}t} \right)$ (2)

$\frac{t}{q_{t}}=\frac{1}{k_{2}\cdot q_{e}^{2}}+\frac{t}{q_{e}}$ (3)

where *q_t_* and *q_e_* represent the amount of atrazine (mg/kg) removed at time *t* and at equilibrium, respectively; k_1_ and k_2_ represent the first-order and second-order adsorption percentage constants (1/h), respectively.

The isothermal adsorption experiments were performed using the Freundlich equation [Eq. (4)] and the Langmuir equation [Eq. (5)] to analyze the adsorption behavior of atrazine by 20 ℃, 25 ℃, and 30 ℃ of carrier materials and immobilized carrier materials.

$q_{e}=K_{f}C_{e}^{n}$, (4)

$q_{e}=\frac{q_{m}{KC}_{e}}{1+{KC}_{e}}$, (5)

where C_e_ is the mass concentration of atrazine in the solution at equilibrium (mg/kg); K*_f_* is the Freundlich equilibrium constant; n is the constant characterizing the adsorption strength; q_m_ is the theoretical maximum adsorption amount; K is the adsorption affinity coefficient (L/mg).

**Table 2.** Box-Behnken design for optimizing the biodegradation of atrazine by immobilized bacterial mixture (IM-FN) on three factors (A, B, and C) at three levels

| **Components (factors)** | **Level of each factor** | | | |
| --- | --- | --- | --- | --- |
|  | **Low (**–**1)** | **Medium (0)** | **High (+1)** |  |
| A: pH | 8 | 9 | 10 |  |
| B: Temperature (℃) | 25 | 30 | 35 |  |
| C: Initial concentration of atrazine (mg/L) | 20 | 60 | 100 |  |

**Table 3.** Box-Behnken design matrix and effects of three different factors on the degradation of atrazine by immobilized bacterial mixture (IM-FN).

| **Level of coded (uncoded) value** | | | | |
| --- | --- | --- | --- | --- |
| **Run** | **A (pH)** | **B (Temperature, ℃)** | **C (****Initial concentration of atrazine, mg/L)** | **Degradation percentage (%)** |
| 1 | 1 (10) | –1 (25) | 0 (60) | 69.35 |
| 2 | 0 (9) | 0 (30) | –1 (20) | 68.56 |
| 3 | 1 (10) | 1 (35) | 0 (60) | 70.89 |
| 4 | 1 (10) | 0 (30) | 1 (100) | 72.81 |
| 5 | –1 (8) | 1 (35) | 0 (60) | 72.25 |
| 6 | –1 (8) | –1 (25) | 0 (60) | 69.39 |
| 7 | 0 (9) | 0 (30) | 0 (60) | 96.69 |
| 8 | 0 (9) | –1 (25) | 1 (100) | 69.64 |
| 9 | 0 (9) | 0 (30) | 0 (60) | 98.59 |
| 10 | 0 (9) | –1 (25) | -1 (20) | 65.92 |
| 11 | 0 (9) | 0 (30) | 0 (60) | 97.56 |
| 12 | 0 (9) | 1 (35) | –1 (20) | 67.68 |
| 13 | 1 (10) | 0 (30) | –1 (20) | 68.29 |
| 14 | 0 (9) | 0 (30) | 0 (60) | 97.82 |
| 15 | 0 (9) | 0 (30) | 0 (60) | 96.64 |
| 16 | 0 (9) | 1 (35) | 1 (100) | 67.21 |
| 17 | 1 (10) | 0 (30) | 1 (100) | 69.16 |

**Table 4.** Analysis of variance of regression model.

| **Source** | **Sum of squares** | **Freedom** | **Mean square** | **F value** | | **P value** | **Significance** |
| --- | --- | --- | --- | --- | --- | --- | --- |
| A | 0.4900 | 1 | 0.4900 | 0.3733 | 0.5605 | |  |
| B | 1.74 | 1 | 1.74 | 1.32 | 0.2875 | |  |
| C | 8.76 | 1 | 8.76 | 6.67 | 0.0363 | |  |
| AB | 0.4356 | 1 | 0.4356 | 0.3319 | 0.5826 | |  |
| AC | 3.84 | 1 | 3.84 | 2.93 | 0.1309 | |  |
| BC | 4.39 | 1 | 4.39 | 3.34 | 0.1102 | |  |
| A^2^ | 652.51 | 1 | 652.51 | 497.12 | < 0.0001 | |  |
| B^2^ | 890.31 | 1 | 890.31 | 678.29 | < 0.0001 | |  |
| C^2^ | 986.45 | 1 | 986.45 | 751.53 | < 0.0001 | |  |
| Model | 2843.39 | 9 | 315.93 | 240.70 | < 0.0001 | | significant |
| Residual | 9.19 | 7 | 1.31 |  |  | |  |
| Lack of Fit | 6.51 | 3 | 2.17 | 3.23 | 0.1432 | | not significant |
| Pure Error | 2.68 | 4 | 0.6704 |  |  | |  |
| Total | 2852.58 | 16 |  |  |  | |  |


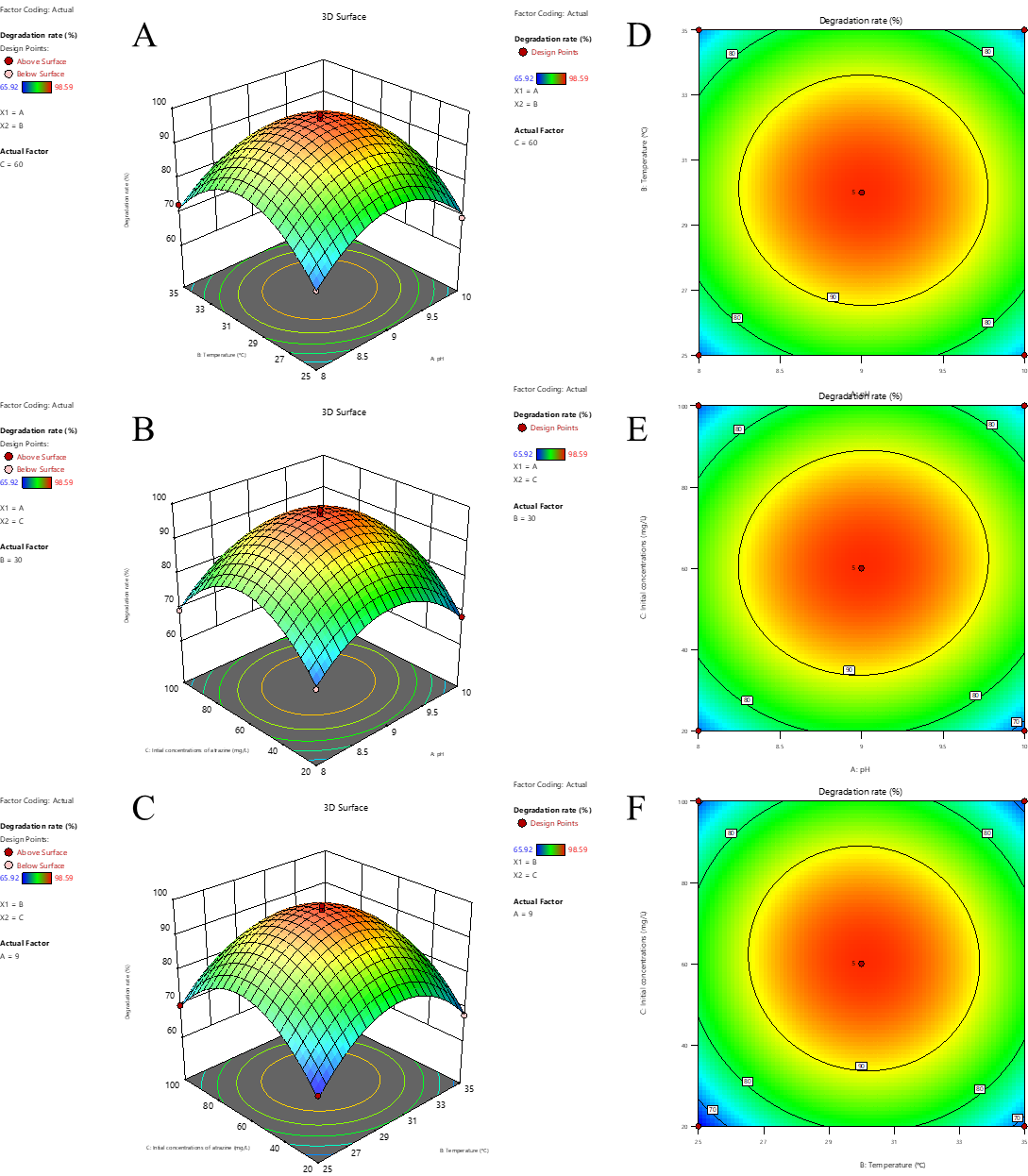


**Figure 1.** Contour plots (**A–C**) and 3D response surface plots (**D–F**) for degradation percentage of atrazine biodegradation based on the optimization of pH and temperature (**A, D**), pH and initial concentration of atrazine (**B, E**), and initial concentration of atrazine and temperature (**C, F**).

**Figure 2.** PCR detection of total soil DNA without strains FH-1 and NJ-1. M: marker; lanes 1 and 2 represent the gel electrophoresis patterns of the gene *PydC* of strain FH-1 encoding the Zn^2+^-dependent hydrolase and the esterase gene *estD* of NJ-1, respectively, showing no amplification from the total DNA of soil.

**Figure 3.** Qualitative determination of immobilized bacterial mixture (IM-FN) and bacterial suspensions (FN) in soil. (**A**) Electrophoresis gel diagram of *Klebsiella variicola* strain FH-1 Zn^2+-^dependent hydrolase coding gene *PydC* amplified from total soil DNA. (**B**) Electrophoresis gel diagram of *Arthrobacter* sp. strain NJ-1esterase coding gene *estD* amplified from total soil DNA. Lane M: Marker; Lanes 1, 3, 5, 7, and 9 represent the bands amplified based on the immobilized bacterial mixture (IM-FN) from total soil DNA on day 1, 3, 5, 7 and 14, respectively; Lanes 2, 4, 6, 8, and 10 represent the bands amplified based on bacterial suspensions (FN) from the total soil DNA on day 1, 3, 5, 7 and 14, respectively.
